# Modulation of Spin Dynamics in 2D Transition‐Metal Dichalcogenide via Strain‐Driven Symmetry Breaking

**DOI:** 10.1002/advs.202200816

**Published:** 2022-05-01

**Authors:** Tao Liu, Du Xiang, Hong Kuan Ng, Zichao Han, Kedar Hippalgaonkar, Ady Suwardi, Jens Martin, Slaven Garaj, Jing Wu

**Affiliations:** ^1^ Institute of Optoelectronics & Zhangjiang Fudan International Innovation Center Fudan University Shanghai 200438 China; ^2^ Frontier Institute of Chip and System & Zhangjiang Fudan International Innovation Center Fudan University Shanghai 200438 China; ^3^ Institute of Materials Research and Engineering Agency for Science, Technology and Research 2 Fusionopolis Way, Innovis, #08‐03 Singapore 138634 Singapore; ^4^ Department of Materials Science and Engineering Nanyang Technological University Singapore 639798 Singapore; ^5^ Leibniz‐Institut für Kristallzüchtung Max Born Str 2 Berlin 12489 Germany; ^6^ Department of Physics National University of Singapore, Singapore Science Drive 3 Singapore 117551 Singapore; ^7^ Department of Biomedical Engineering National University of Singapore 4 Engineering Drive 3 Singapore 117583 Singapore; ^8^ Department of Materials Science and Engineering National University of Singapore 9 Engineering Drive 1 Singapore Singapore 117575 Singapore

**Keywords:** spin–orbit splitting, spin–strain coupling, strain engineering, transition metal dichalcogenides, weak antilocalization

## Abstract

Transition metal dichalcogenides (TMDs) possess intrinsic spin–orbit interaction (SOI) with high potential to be exploited for various quantum phenomena. SOI allows the manipulation of spin degree of freedom by controlling the carrier's orbital motion via mechanical strain. Here, strain modulated spin dynamics in bilayer MoS_2_ field‐effect transistors (FETs) fabricated on crested substrates are demonstrated. Weak antilocalization (WAL) is observed at moderate carrier concentrations, indicating additional spin relaxation path caused by strain fields arising from substrate crests. The spin lifetime is found to be inversely proportional to the momentum relaxation time, which follows the Dyakonov–Perel spin relaxation mechanism. Moreover, the spin–orbit splitting is obtained as 37.5 ± 1.4 meV, an order of magnitude larger than the theoretical prediction for monolayer MoS_2_, suggesting the strain enhanced spin‐lattice coupling. The work demonstrates strain engineering as a promising approach to manipulate spin degree of freedom toward new functional quantum devices.

## Graphical abstract

Strain driven symmetry breaking induces additional spin relaxation path in bilayer MoS_2_, enabling weak antilocalization effect under moderate electric field. The extracted spinorbit splitting energy is 37.5 ± 1.4 meV, an order of magnitude larger than that predicted for monolayer MoS_2_. These findings demonstrate strain engineering as a reliable approach for spin manipulation in 2D transition metal dichalcogenides.

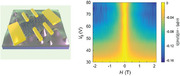

## 1 Introduction

Spin–orbit interaction (SOI), originating from the spin‐momentum coupling of a particle under an external electric potential, plays a crucial role in the application of spin‐based quantum information technology and spintronics.^[^ ^1^, ^2^, ^3^, ^4^, ^5^ ^]^ In the absence of inversion symmetry, the spin degeneracy lifted by SOI induces exotic spin dynamics, such as spin ballistic transport and spin Hall effect.^[^ ^6^, ^7^ ^]^ The spin polarization is determined by the orientations of momentum **p** and electric field **E** as described by the SOI Hamiltonian ${\hat{H}}_{\mathrm{so}}=\;-\mu_{B}\sigma \cdot B_{\mathrm{eff}}$, with the effective magnetic field **B**_eff_ = (**p** × **E**/2*mc* ^2^) , where *μ*_B_, *σ*, *m*, and *c* are the Bohr magneton, spin Pauli matrices, carrier effective mass, and light velocity, respectively.^[^ ^8^ ^]^ In most semiconductor systems, an external electric field is used to modulate **B**_eff_, leading to a Rashba‐type spin splitting with **E**‐dependent in‐plane spin polarization.^[^ ^9^, ^10^, ^11^ ^]^

The emerging 2D transition metal dichalcogenides (TMDs) exhibit intrinsic SOI, which is very promising for spintronic applications.^[^ ^4^, ^5^, ^12^ ^]^ However, the manipulation of **B**_eff_ in these materials often requires large electric fields, which is generally not accessible by the single (back‐) gate device geometry and can only be produced so far by ion gel gating or dual gating, complicating the practicality of these devices.^[^ ^13^, ^14^ ^]^ More importantly, the requirement of inversion asymmetry for spin–orbit splitting restricts 2D TMDs spintronics to only odd number of layers.^[^ ^15^, ^16^ ^]^

Theoretically, in the presence of SOI, manipulation of the spin degree of freedom of carriers is possible by directly controlling the orbital motion, which in turn can be realized through deformations of the crystal lattice.^[^ ^17^ ^]^ Owning to the atomic thickness of 2D TMDs, such lattice deformations could be effectively achieved by the strain fields arising from nano‐/micro‐scale ripples, wrinkles, and topological disorder (grain boundaries) in these materials.^[^ ^18^, ^19^, ^20^ ^]^

In this paper, we demonstrate strain field modulated spin dynamics using crested bilayer molybdenum disulfide (MoS_2_). We employ bilayer MoS_2_ because of its zero intrinsic spin–orbit splitting due to symmetry considerations.^[^ ^21^ ^]^ The crested MoS_2_ (c‐MoS_2_) device is fabricated on a silicon nitride (SiN*_x_*) substrate with high surface corrugation, where the root‐mean‐square (RMS) roughness *δ*Z_RMS_ is about 2.0 nm.^[^ ^22^ ^]^ We observe weak antilocalization (WAL) in magnetoconductance at relatively low carrier concentration. The Hikami‐Larkin‐Nagaoka (HLN) localization theory and Hall effect measurements are used to extract the spin relaxation time *τ*_*so*_ and the momentum relaxation time *τ*_*p*_, respectively. Our results show that *τ*_*so*_ is inversely proportional to *τ*_*p*_, suggesting the Dyakonov–Perel (DP) relaxation mechanism. Using a generic **k · p** model for strained TMDs, the spin orbit splitting energy *ε*_*z*_ is determined as 37.5 ± 1.4 meV, demonstrating an order of magnitude enhancement comparing to the intrinsic value of the standard monolayer counterpart (3 meV).^[^ ^23^ ^]^ Moreover, we find that the dominating factor for phase coherence in our system is electron‐phonon interaction, instead of electron‐electron interaction, indicating strain‐induced modulation of the scattering processes. Our approach envisions strain engineering as a novel and effective method to modulate the spin dynamics in 2D TMDs, not limited by inversion symmetry considerations and hence applicable to all multi‐layer devices.

## 2 Results

### 2.1 Electrical and Magneto Transport Characterization of c‐MoS2 Device

**Figure 1:**
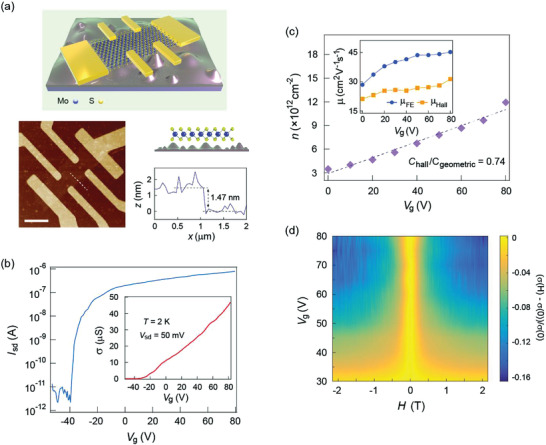
a) Schematic of c‐MoS_2_ device in a Hall bar structure. The AFM image demonstrates the bilayer nature of the MoS_2_ flake. Scale bar is 4 µm. b) Transfer curve measured at *T* = 2 K with two‐probe configuration. Inset shows the conductivity of the device. c) Gate dependent carrier concentration extracted from Hall effect measurements at *T* = 2 K. Field effect mobility and Hall mobility are depicted in the inset. The ratio between Hall capacitance and geometric capacitance is determined as 0.74. d) Normalized magnetoconductance as a function of gate voltage at *T* = 2 K. The initial measured curves were symmetrized as *σ*(*H*) = [*σ*(+*H*) + *σ*(−*H*)]/2 to avoid contributions from the sample geometry.

First, we investigate the electrical transport properties of the bilayer c‐MoS_2_. **Figure** **1**a shows the schematic of the as‐fabricated c‐MoS_2_ device in a Hall‐bar structure. The atomic force microscope (AFM) image reveals the thickness of the flake as 1.47 nm, demonstrating its bilayer nature which is consistent with the Raman spectrum (Figure S1, Supporting Information). The sheet conductivity *σ* as a function of gate voltage *V*_g_ shows typical n‐type transport behaviour (Figure 1b). The temperature‐dependent transport characterization (Figure S2, Supporting Information) indicates a metal‐insulator transition at relatively high gate voltage (*V*_g_ > 65 V) compared to other MoS_2_ devices.^[^ ^24^, ^25^ ^]^ We attribute this to the surface crests induced potential traps in the MoS_2_ channel, in which a larger electric field is required to prompt the system into the diffusive regime.

The Hall coefficient *R*_H_ at multiple gate voltages is extracted from Hall effect measurements (Figure S3, Supporting Information) and the sheet carrier density *n* is determined using *n* = 1/(*eR*_H_), where *e* is the elementary charge (Figure 1c). The carrier density increases linearly with the gate voltage, ranging from 3 × 10^12^ cm^–2^ (*V*_g_ = 0 V) to 1.2 × 10^13^ cm^–3^ (*V*_g_ = 80 V), consistent with those reported in similar material systems.^[^ ^25^, ^26^ ^]^ The gate‐dependent field effect mobility *μ*_FE_ and Hall mobility *μ*_Hall_ are depicted in the inset of Figure 1c, where *μ*_FE_ is larger than *μ*_Hall_ as observed in previous reports.^[^ ^22^, ^28^ ^]^ We note that the crested interface could influence the capacitive coupling between the MoS_2_ flake and the substrate, which may lead to the under/overestimation of the field effect mobility. Therefore, we extract the device capacitance *C*_hall_ from the Hall effect measurements. The *C*_hall_ is determined to be in agreement with the geometric capacitance *C*_geometric_ used for calculating the field effect mobility, illustrating the reliability of the *μ*_FE_ in our experiment (Figure 1c).

Now, we focus on magneto‐transport measurements to probe the SOI in response to a strain field. An overview of our experimental results at 2 K is shown in Figure 1d, demonstrating the dependence of the normalized magnetoconductance (MC), (*σ*(H) – *σ*(0))/*σ*(0), where *σ*(0) is the zero‐field conductance, on charge carrier density. While no significant MC is detected at low gate voltage, we observe negative MC for *V*_g_ > 30 V, indicating prevailing weak anti‐localization (WAL). This is in contrast to previous reports of similar material systems, in which weak localization (WL) starts at weak field, followed by a possible crossover to WAL as the magnetic field is increased under sufficiently high charge carrier concentration.^[^ ^13^, ^27^, ^29^ ^]^ In our c‐MoS_2_ devices, carrier concentration in the channel remains in the order of 10^12^ cm^–2^, far below the criteria of electrostatic modulation of WAL (≈5 × 10^13^ cm^–2^).^[^ ^30^ ^]^ Moreover, bilayer MoS_2_ devices which we fabricated with the same processing steps on standard SiO_2_ (*δ*Z_RMS_ ≈ 0.18 nm) and smooth SiN*_x_* substrates (*δ*Z_RMS_ ≈ 0.4 nm) show no detectable magnetoconductance within the experimental regime (Figure S4a,b, Supporting Information), implying a relatively high disorder level in these devices. On the other hand, we measured multiple devices with varied sample thicknesses ranging from monolayer to 5‐layer, where clear WAL signals are identified regardless of the layer number, suggesting that such phenomenon is not specific to a particular sample geometry with typical symmetry (Figures S5 and S6a, Supporting Information). We note that all our devices were not exposed to a post‐fabrication annealing processes as often described in previous reports.^[^ ^25^, ^31^, ^32^ ^]^ Therefore, the occurrence of WAL in crested devices indicates that the substrate morphology could play a vital role in determining the interference in 2D‐devices. Specifically, it may allow us to identify WAL in the strained regions of crested devices.

### 2.2 Investigation of Magnetotransport via HLN Localization Theory

**Figure 2:**
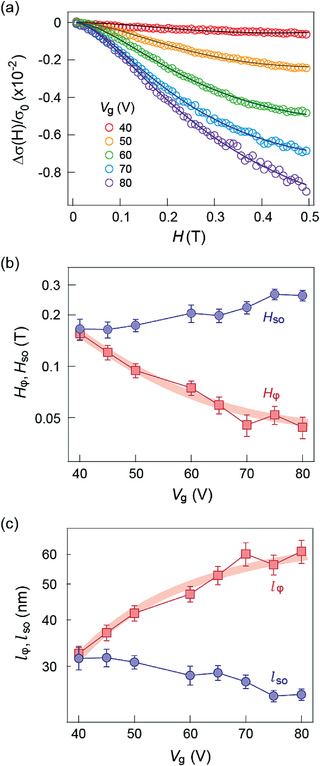
a) Magnetoconductance Δ*σ*/*σ*_0_ fitting using the HLN equation. Δ*σ* = *σ*(H) – *σ*(0). *σ*_0_ = *e* ^2^/*π*ℏ is a universal value of quantum conductance. Experimental results are indicated by circles, and the solid lines are the results of theoretical fitting. b) Extracted fitting parameters *H*_*ϕ*_ and *H_so_* as a function of carrier concentration *n*. c) Phase coherence length *l*_*ϕ*_ and spin relaxation length *l_so_* deduced from *H*_*ϕ*_ and *H_so_*. Bold red curves in (b) and (c) are guides to eyes.

To understand the relationship of strain with the SOI, we analyze the observed magnetoconductance using the Hikami‐Larkin‐Nagaoka (HLN) localization theory^[^ ^33^ ^]^

(1) $$\begin{array}{c} \Delta \sigma \;=\frac{e^{2}}{2\pi^{2}\hbar }\;\left[F\left(\frac{H}{H_{\varphi }+H_{so}}\right)+\frac{1}{2}F\left[\frac{H}{H_{\varphi }+2H_{so}}\right]-\frac{1}{2}F\left[\frac{H}{H_{\varphi }}\right]\right] \end{array}$$

where *F*(*z*) is defined as *F*(*z*) = *ln*(*z*) + *ψ*(1/2+1/*z*) with *ψ* as the digamma function, and ℏ is the reduced Planck constant. Here, the system is parameterized by only two terms of the characteristic magnetic fields, $H_{\varphi }=\;\hbar /\left(4el_{\varphi }^{2}\right)$ and $H_{\mathrm{so}}=\;\hbar /\left(4el_{\mathrm{so}}^{2}\right)$, where *l*_*ϕ*_ = (*Dτ*_*ϕ*_)^1/2^ is the phase coherence length, *l*_so_ = (*Dτ*_so_)^1/2^ is the spin relaxation length, and D is the diffusion constant. *τ*_*ϕ*_ and *τ*_*so*_ measure the dephasing time and the spin relaxation time, respectively. We perform fitting in the range of 0.5 T to avoid possible contributions from classic MC.^[^ ^14^ ^]^ As shown in **Figure** **2**a, our experimental data demonstrate a good agreement with the HLN theory, particularly at high *V*_g_. We note that the observed amplitude of WAL in our experiment is suppressed by roughly an order of magnitude. We therefore re‐scale the standard HLN theory to fit the experimental curve.^[^ ^34^ ^]^ The suppression could be attributed to the mixture of strained and nonstrained parts of the sample, in which we expect WAL only in the strained areas while the unstrained parts would show zero magnetoconductance similar to our standard devices fabricated on SiO_2_ and smooth SiN*_x_* (Figure S4, Supporting Information). Due to the partial coverage of strained regions, an overall suppression of WAL is therefore expected.

According to Figure 2b, the extracted *H*_*ϕ*_ decreases and *H*_so_ increases with increasing carrier concentration (larger *V*_g_), implying the electric field tunable SOI and spin precision, consistent with the more pronounced WAL peak at higher *V*_g_. The *V*_g_ dependence of *l*_*ϕ*_ and *l*_so_ is demonstrated in Figure 2c. As *V*_g_ increases from 40 V (*n* = 6.7 × 10^12^ cm^–2^) to 80 V (*n* = 1.2 × 10^13^ cm^–2^), a monotonic increase of *l*_*ϕ*_ from 32 to 61 nm is observed, while *l_so_* decreases from 31 to 25 nm. The relationship of the length scale *l*_so_ < *l*_*ϕ*_ at *V*_g_ > 40 V provides direct evidence of the WAL regime. Additionally, the absence of any crossover between *l*_so_ and *l*_*ϕ*_ implies no WL‐WAL transition in the system, consistent with our experimental observation.

### 2.3 Strain Induced Enhancement in the Spin‐Orbit Splitting

The observed WAL scenario is similar to that expected in a system with strong electric field induced Rashba spin splitting. In such a system, intervalley scattering is considered as an inelastic scattering process and associated with intervalley phonons,^[^ ^35^ ^]^ where the scattering rate is governed by the long‐range electron‐phonon (e–p) exchange interactions.^[^ ^36^, ^37^ ^]^ The electrostatic doping can induce screening effect that effectively suppress such interactions, giving rise to the decrease of the intervalley scattering rate.^[^ ^38^, ^39^ ^]^ However, in a transistor with conventional (back‐) gate geometry, such high electric field is unexpected unless in ion gel gating or dual gating. The existence of substrate crests in our devices considerably increases the density of random strain fluctuations, which are long‐ranged potential that introduces disorder to increase intravalley scattering over intervalley scattering.^[^ ^40^ ^]^ While this helps to reduce the intervalley scattering rate for WAL to take place, it also means that an additional spin relaxation path of charge carriers is necessary to maintain the required limit of *l*_so_ < *l*_*ϕ*_.

It has been demonstrated that spin relaxation of carriers in 2D TMDs can be caused by topographic deformations induced by a local strain field (for example, static wrinkles), even in the crystals that are intrinsically free of defects.^[^ ^23^ ^]^ For monolayer TMDs, this is due to the direct coupling of spin and strain at the *K* point, which allows spin relaxation when translation symmetry in the system is broken, for example, by the existence of ripples.^[^ ^17^ ^]^ We note that for few layer TMDs, several calculations suggest that the conduction band minimum (CBM) is located at *Q* point instead of *K* point.^[^ ^41^, ^42^ ^]^ However, the CBM can be shifted back to *K* point by applying any nonzero tensile strain.^[^ ^43^ ^]^ Due to the spike‐like feature of the substrate's morphology, tensile strain is dominating in our device, hence the *K*‐valley descriptions can be safely adopted.

**Figure 3:**
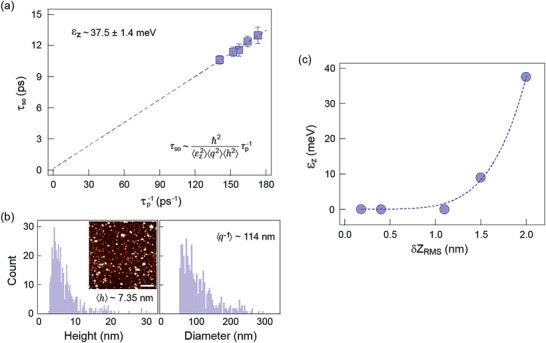
a) *τ*_*so*_ as a function of $\tau_{p}^{-1}$. The blue dashed line is the extrapolation of the linear fitting. The relationship $\tau_{so}\propto \tau_{p}^{-1}$ indicates the DP dominated spin relaxation. The *ε*_*z*_ is calculated as 37.5 ± 1.4 meV. b) The statistical distributions of the lateral size and height of substrate crests in a 5 µm × 5 µm area, with <*h*> and <*q* ^−1^> determined as 7.35 and 114 nm, respectively. The inset of b) shows the corresponding AFM image. Scale bar is 1 µm. c) The spin splitting energy as a function of the substrate roughnesses. A monotonic increase in the *ε*_*z*_ is observed as increasing the *δZ_RMS_*. The blue dashed line is a fit with a power function to have a better visualization of the correspondence between the two parameters.

In our experiment, the substrate induced lattice deformations breaks the local z→−z mirror symmetry even for bilayer MoS_2_, coupling the in‐plane spin components *s_x_*_,_ *_y_* of the electron to its lateral orbital motion. This breaking of mirror symmetry thus generates a possible spin relaxation path through spin‐lattice coupling. To validate this scenario, we analyze the spin relaxation mechanism using a generic **k · p** model developed for 2D hexagonal crystals (2DHCs).^[^ ^23^ ^]^ This model describes the spin dynamics in a 2D system with static wrinkles, which exactly matches our experimental conditions and is utilized as a reliable method for the quantitative estimation of the spin relaxation. The 2DHC is regarded as a flexible membrane, with *q* ^–1^ and $\sqrt{\left\langle h^{2}\right\rangle }$ representing the lateral size and height of the static wrinkles, respectively. In **Figure** **3**a, *τ*_*so*_ is revealed to be inversely proportional to the momentum relaxation time *τ*_*p*_ with *τ*_*so*_ ≫ *τ*_*p*_, indicating that the system follows a Dyakonov–Perel (DP) spin relaxation mechanism.^[^ ^13^ ^]^ To exclude the possible contributions from classical artefacts, the *τ*_*p*_ is determined using the mobility and concentration obtained from the Hall measurements. Referring to the **k · p** model, in the DP dominated regime, we have $\tau_{so}\;\approx \;\hbar^{2}/\left(\varepsilon_{z}^{2}\left\langle q^{2}\right\rangle \left\langle h^{2}\right\rangle \tau_{p}\right)$, where *ε*_*z*_ determines the spin‐lattice coupling strength and the intrinsic spin–orbit splitting. To obtain the effective size of the substrate crests, we analyze the topography of the crested substrate using AFM (inset of Figure 3b). The statistical distributions of the spike size are demonstrated in Figure 3b, where the average values <*h*> and <*q* ^−1^> are determined as 7.35 and 114 nm, respectively. These two parameters determine the equivalent spike size over a mesoscopic device scale, serving as reasonable quantifications of the effective strain field experienced by the device. The value of *ε*_*z*_ is then calculated to be 37.5 ± 1.4 meV, which is an order of magnitude larger than that of the theoretical prediction of monolayer MoS_2_ conduction band (3 meV).^[^ ^23^, ^44^ ^]^

We make detailed comparisons between our results and the previous works in both theoretical and experimental aspects (**Table** **1**).^[^ ^13^, ^45^, ^46^, ^47^, ^48^ ^]^ The strain engineered MoS_2_ device demonstrates a significant enhancement of the spin splitting energy with a simplified device structure, which is much more superior to the previous works. We note that only monolayer cases in previous reports have been included since the intrinsic value for bilayer MoS_2_ is expected to be zero due to the inversion symmetry.^[^ ^49^ ^]^ These results suggest that the out‐of‐plane lattice deformation induced strain field could enhance the spin‐lattice coupling, leading to additional spin relaxation of carriers that propel the system into WAL regime. We also fabricated monolayer devices to further confirm our observations, where intrinsically breaking of inversion symmetry exists. Similar WAL signals were observed with consistent scaling of the relevant length scales, demonstrating the strain induced spin dynamics modulation (Figure S6b,c, Supporting Information). The spin splitting energy in such a crested monolayer device was determined as *ε*_*z*_ = 38.2 ± 2.7 meV (Figure S6c, Supporting Information), which is comparable to the value of bilayer device. These results provide stronger evidence on the strain induced enhancement in the SOI that gives rise to a larger spin splitting.

**Table 1:** Comparison of the splitting/spacing energy *ε*_*z*_ in MoS_2_ between our work and previous reports

Ref.	mode	thickness	structure	****ε****_***z ***_ [meV]
^[^ ^45^ ^]^	theory	1‐layer	‐	3‐4
^[^ ^46^ ^]^	theory	1‐layer	‐	3
^[^ ^47^ ^]^	theory	1‐layer	‐	3
^[^ ^13^ ^]^	experiment	1‐layer	dual‐gate	4.3 ± 0.1
^[^ ^48^ ^]^	experiment	1‐layer	dual‐gate	0.8‐2.0
This work	experiment	2‐layer	back‐gate	37.5 ± 1.4

**Figure 4:**
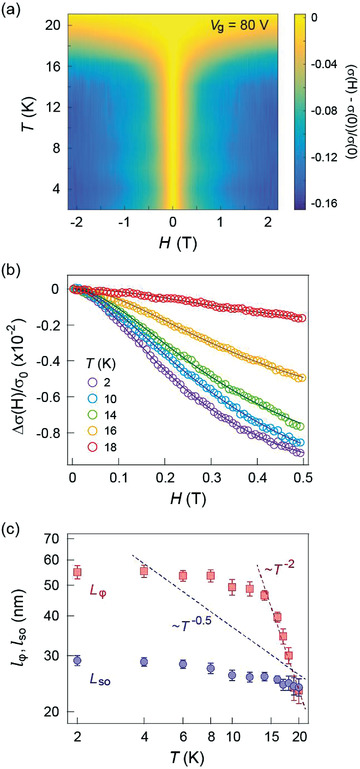
a) Temperature dependence of the magnetoconductance at *V*_g_ = 80 V. WAL is observed at *T* < 20 K. The *σ*(H) was also symmetrized as *σ*(H) = [*σ*(+H) + *σ*(‐H)]/2. b) Magnetoconductance Δ*σ*/*σ*_0_ fitting using the HLN equation. Experimental results are indicated by circles, and the solid lines are the results of theoretical fitting. c) Temperature dependence of *l*_*ϕ*_ and *l_so_* at *V*_g_ = 80 V. The dashed red line shows agreement with *T* ^−2^ dependence at *T* > 14 K. The dashed blue line indicates the electron‐electron interaction that follows *T* ^−0.5^.

To have a more quantitative measure on the spin–strain coupling, we employed the wet polishing method to smooth the corrugated SiN*_x_* film, then fabricated the bilayer devices on smoothed substrates with different *δZ_RMS_* (1.5 and 1.1 nm, respectively). The AFM images of all the relevant substrate morphologies included in our work are shown in Figure S7 in the Supporting Information. We then conducted magneto‐transport measurements on these devices (Figures S4c and S8a, Supporting Information). The device with *δZ_RMS_* = 1.1 nm shows no detectable WAL signal, similar to those fabricated on the standard SiO_2_ and the smooth SiN*_x_* substrates (Figure S4a, Supporting Information; **Figure** **4**b). This could be due to the insufficient strain field generated by such surface morphology, or the signal is too weak to be detected within our experimental resolution. Therefore, we assign the spin splitting energy to be zero in the devices with no WAL signal. For the device with *δZ_RMS_* = 1.5 nm, the length scales show the relationship of *l_so_* < *l*_*ϕ*_, which confirms the presence of the WAL effect (Figure S8b, Supporting Information). On the other hand, in terms of the time scales, the *τ*_*so*_ is proportional to the 1/*τ*_*p*_ with *τ*_*so*_ ≫ *τ*_*p*_, indicating the same DP spin relaxation mechanism as that shown in Figure 3a (Figure S8c, Supporting Information). The spin–orbit splitting energy *ε*_*z*_ is determined as 9.0 ± 1.5 meV using the same **k · p** model (Figure S8c, Supporting Information). By summarising the *ε*_*z*_ as a function of the substrates *δZ_RMS_*, we obtained a monotonically increasing trend between these two parameters (Figure 3c), suggesting that the substrate corrugation induced strain field can be utilized to controllably modulate the spin dynamics in a 2D system. It is worth noting that such spike induced strain gradient discussed here is significantly different from the homogeneous strain field produced by the global in‐plane “pulling/stretching”, where the later does not break the inversion symmetry of a 2D system.^[^ ^50^, ^51^ ^]^

### 2.4 Temperature‐Dependent Magmetotransport Characterization

Finally, we analyze the temperature dependence in our system to investigate the effect of the strain field on the dephasing process. Figure 4a shows the temperature dependence of the normalized magnetoconductance at *V*_g_ = 80 V, where WAL is observed for temperatures *T* < 20 K. The MC shows excellent agreement with HLN theory at weak magnetic fields, enabling us to extract the relevant length scales. The extracted *l*_*ϕ*_ and *l*_so_ are depicted in Figure 4c. It is worth noting that while no significant variation of the experimental MC is observed at *T* < 14 K, WAL rapidly collapses when temperature increases from 14 to 20 K. This rather abrupt transition implies a different dominating dephasing mechanism in the system, rather than the common electron‐electron interaction within this temperature regime. While *l_so_* demonstrates weak temperature dependence as expected, *l*_*ϕ*_ shows a saturation behaviour at *T* < 14 K followed by a rapid decrease with increasing temperature. For electron‐electron interactions in 2D systems, we expect a *T* ^–1/2^ dependence for *l*_*ϕ*_,^[^ ^52^ ^]^ which is clearly too shallow with respect to our observation (Figure 4c), whereas electron‐phonon scattering is predicted to have a *T* ^–3/2^ dependence.^[^ ^53^, ^54^ ^]^ Our data is best fitted with *T* ^–2^ which is still consistent with electron‐phonon scattering, therefore suggesting it as the dominating mechanism limiting the phase coherence in this regime. These results further support the scenario that wrinkle‐induced strain fields strongly interact with electronic degrees of freedom, which in turn modify the interference in the system.

## 3 Conclusion

In summary, we have demonstrated the SOI in bilayer MoS_2_ in response to an inhomogeneous strain field. The observed WAL in this system implies an additional spin relaxation path induced by the static wrinkles on the crested SiN*_x_* surface, which is further supported by the order of magnitude enhancement in the strength of spin orbit splitting under strain. The relationship between *τ*_*so*_ and *τ*_*p*_ demonstrates a clear signature of DP dominated spin relaxation, consistent with those reported in 2D semiconductors. Moreover, the phase coherence length is found to be governed by electron‐phonon interaction, indicating that such interaction becomes more relevant at low temperature under strain. Our work reveals strain as a new functionality to enhance the SOC strength and introduce significant spin orbit splitting in 2D materials that originally possess intrinsic inversion symmetry. This could lift the spin degeneracy and increase the charge‐to‐spin conversion efficiency in a 2D system, which in turn provides opportunities for realizing new functional quantum devices.

## 4 Experimental Section

### Device Fabrication and Measurement

MoS_2_ flakes were mechanically exfoliated from bulk MoS_2_ crystals (HQ graphene) onto 300 nm c‐SiN*_x_* (Cornell), 300 nm SiO_2_ (Nova), and 300 nm smooth SiN*_x_* (SVM) substrates. Standard electron‐beam lithography (FEI nano SEM 230) was used to pattern the contacts in a Hall bar structure, followed by thermal evaporation (Kurt J. Lesker, Nano36) of 80 nm gold as electrodes. The electrical characterization, Hall effect measurements, and the magnetotransport were all measured using a Physical Property Measurement System (PPMS, Quantum Design). The devices were mounted on a chip carrier using conductive silver paint. Aluminium wires (with diameter ≈25 µm) were then used to connect the patterned electrode‐pads to the pins on the chip carrier, which was completed using a grounded wire‐bonder setup (F&K Delvotec Model).

### Wet Polishing of SiN*_x_* substrate

To partially remove the surface spikes, we soaked the corrugated SiN*_x_*/Si substrates in hot phosphoric acid with the concentration of 85% at 170 °C. The soaking time was set as 1 and 2 h to achieve the surface roughnesses of 1.5 and 1.1 nm, respectively.

## Conflict of Interest

The authors declare no conflict of interest.

## Supporting information

Supporting Information

## Data Availability

The data that support the findings of this study are available from the corresponding author upon reasonable request.
