# Supplementary material for: Modulation of Spin Dynamics in 2D Transition‐Metal Dichalcogenide via Strain‐Driven Symmetry Breaking
Source: Adv Sci (Weinh). 2022 May 1;9(20):2200816. doi: 10.1002/advs.202200816 (PMC9284128; doi:10.1002/advs.202200816)
Supplement: Supplementary file 1 — Supporting Information [file ADVS-9-2200816-s001.pdf]

Supporting Information

Modulation of spin dynamics in two-dimensional transition-metal dichalcogenide via strain-driven symmetry breaking

Tao Liu, Du Xiang, Hong Kuan Ng, Zichao Han, Kedar Hippalgaonkar, Ady Suwardi, Jens Martin, Slaven Garaj,* Jing Wu**

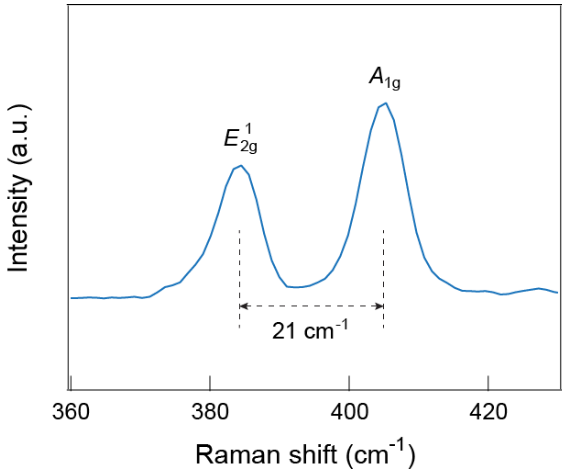

Figure S1. Raman spectrum of the device shown in Figure 1 in the manuscript. Two characteristic peaks at 384 cm^{-1} (E^1_{2g}) and 405 cm^{-1} (A_{1g}) are indicated with a separation of 21 cm^{-1}, confirming its bilayer nature.

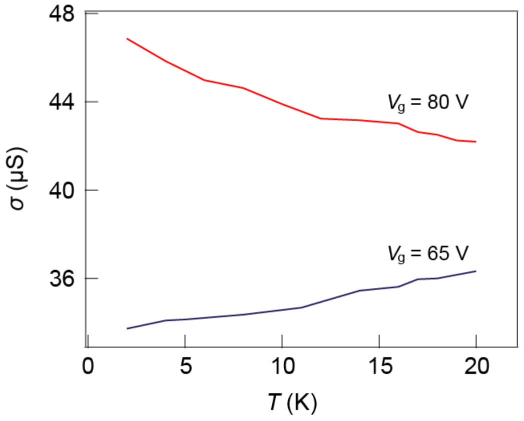

Figure S2. Temperature-dependent conductivity from 2 K to 20 K at V_g = 80 V and 65 V. At V_g = 65 V, the conductivity increases with increasing temperature, indicating a semicunducting behaviour. While at V_g = 80 V, lower conductivity is observed at elevated temperature, demonstrating the existence of metal-insulator transition at V_g > 65 V.

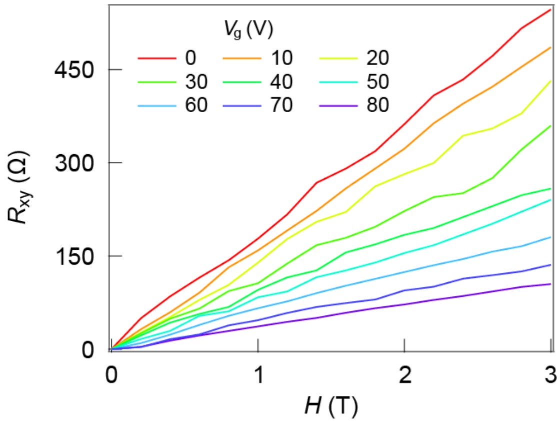

Figure S3. Hall effect measurements at T = 2 K from V_g = 0 V to 80 V.

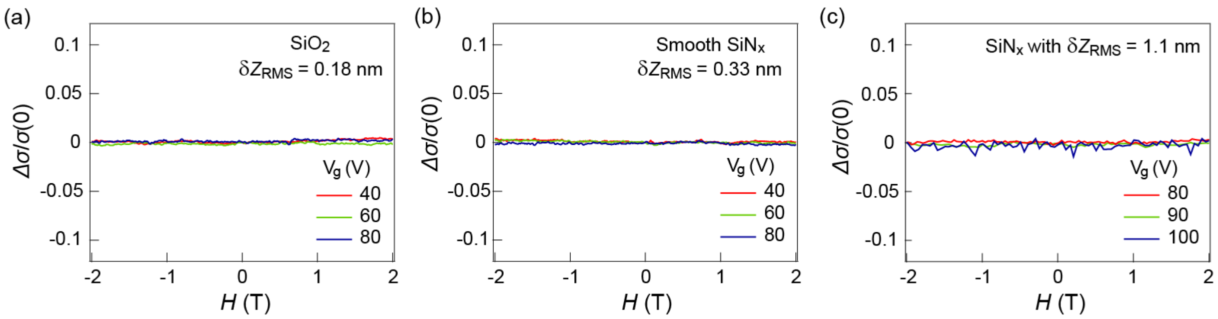

Figure S4. Magnetotransport characterization of bilayer MoS_2 devices on **(a)** standard SiO_2, **(b)** smooth SiN_x, and **(c)** SiN_x with δZ_{RMS} = 1.1 nm, respectively. No magnetoconductance was detected in all the devices within our experimental regime, therefore, we assign the spin splitting energy to be zero in these devices. All the measurements were conducted at T = 2 K.

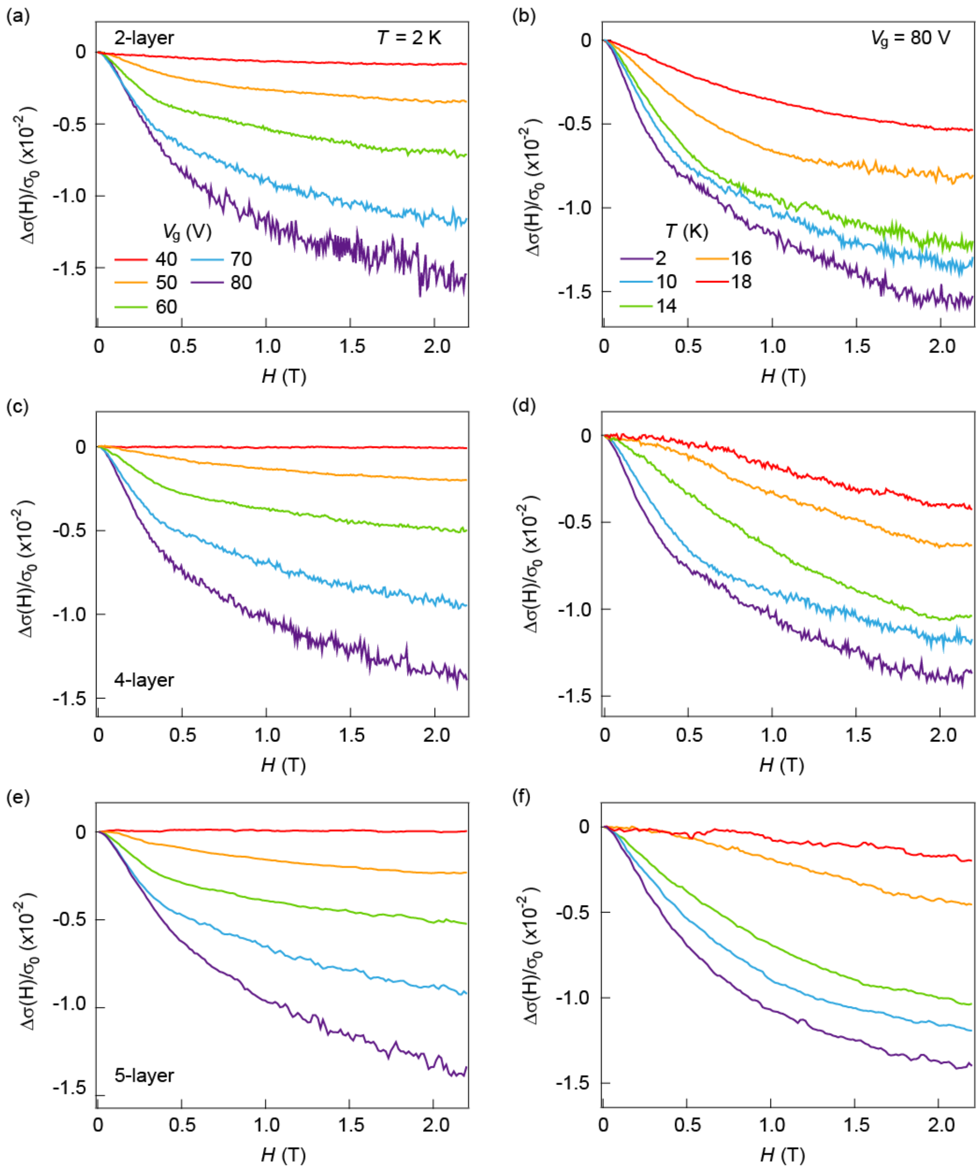

Figure S5. Magneto-transport measurements of different devices with same back-gate geometry. **(a)**, **(c)**, and **(e)** show the gate-dependent WAL signals for 2-, 4-, and 5-layer devices at 2 K, respectively. The corresponding temperature dependence of WAL measured at V_g = 80 V are shown in **(b)**, **(d)**, and **(f)**, respectively. All the devices demonstrate clear WAL signals with similar gate and temperature dependence as that shown in the manuscript, indicating the repeatability of our experiments on different samples.

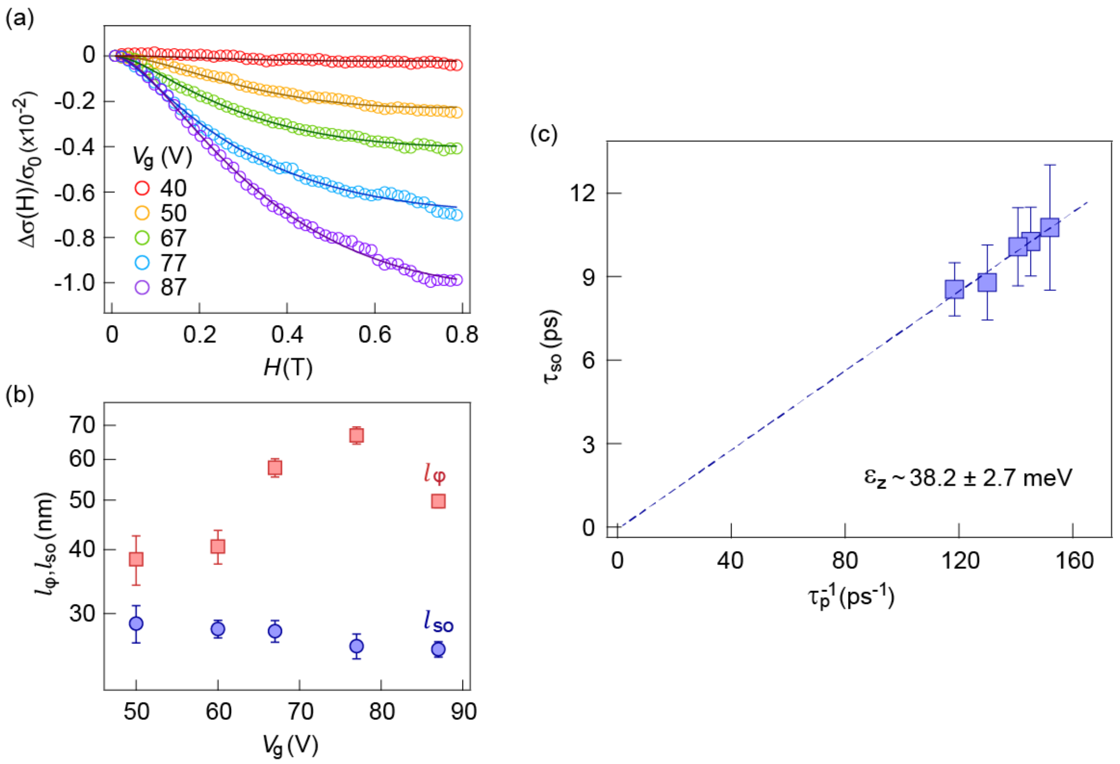

Figure S6. (a) Magnetoconductance $\Delta\sigma/\sigma_0$ of a monolayer MoS_2 device fabricated on the crested substrate. **(b)** Extracted phase coherence length l_{φ} and spin relaxation length l_{so} as a function of V_g, with the relationship $l_{so} < l_{\varphi}$. **(c)** τ_{so} as a function of τ_p^{-1}. The blue dashed line is the extrapolation of the linear fitting. The relationship $\tau_{so} \propto \tau_p^{-1}$ indicates the DP dominated spin relaxation. The ε_z is calculated as 38.2 ± 2.7 meV.

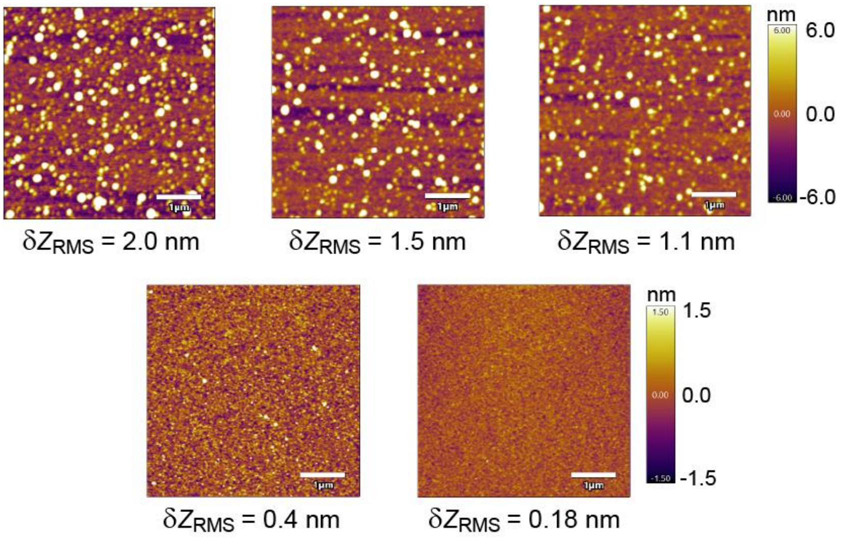

Figure S7. AFM images of all the relevant substrate morphologies used in this work.

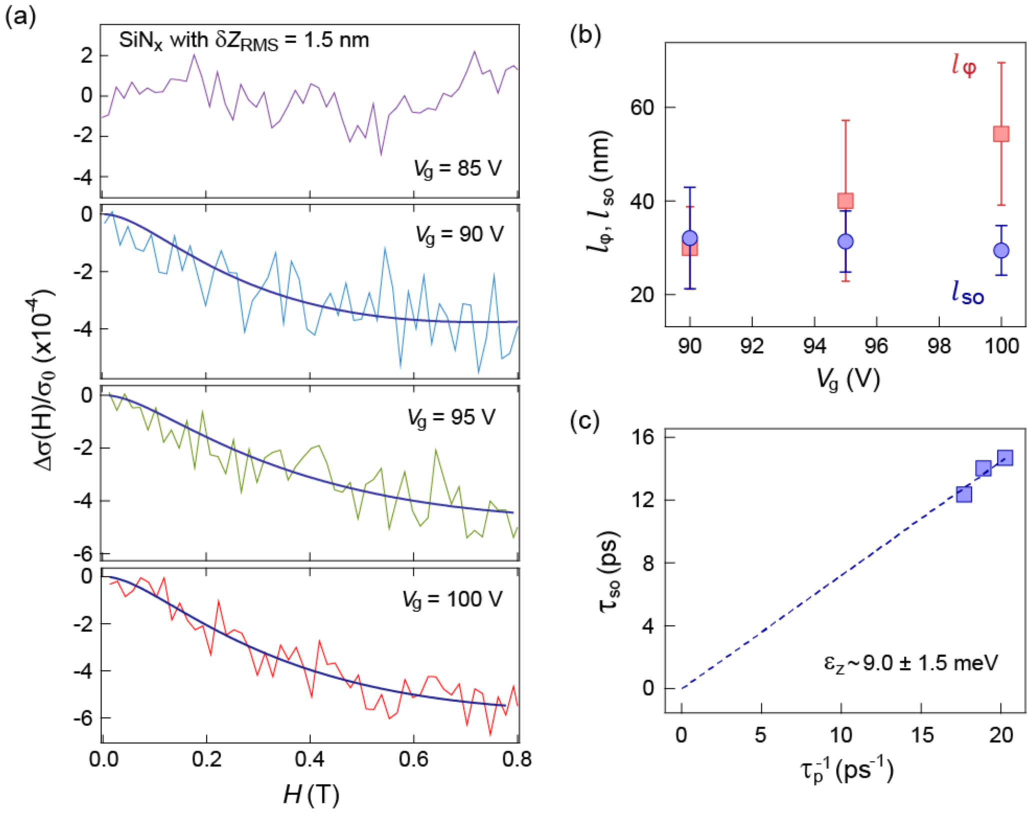

Figure S8. (a) MR measurements of a bilayer MoS_2 device on 1.5 nm corrugate substrate. **(b)** Extracted phase coherence length l_{φ} and spin relaxation length l_{so}, with the relationship of $l_{so} < l_{\varphi}$. **(c)** τ_{so} as a function of τ_p^{-1}. The blue dashed line is the extrapolation of the linear fitting. The relationship of $\tau_{so} \propto \tau_p^{-1}$ indicates the DP dominated spin relaxation. The ε_z is calculated as 9.0 ± 1.5 meV.
